# Attribution or empathy? A study on the public opinion response framework of government social media—a qualitative comparative analysis of 21 public opinion incidents

**DOI:** 10.3389/fpsyg.2025.1556030

**Published:** 2025-07-04

**Authors:** Yuan Li, Mingyang Liu

**Affiliations:** School of Journalism and Communication, Shandong University, Jinan, China

**Keywords:** government social media, public opinion response framework, public opinion ecosystem governance, qualitative comparative analysis, government public opinion response

## Abstract

This study examined the influencing factors of government social media’s public opinion response framework from the perspective of public opinion ecological governance, and provides an optimization strategy for its response. According to a qualitative comparative analysis (fsQCA) of 21 public opinion incidents, it was found that government social media tended to employ a context-responsibility framework when faced with the combined paths of high netizen attention and strong negative sentiment, as well as high media participation and elevated levels of government intervention. In contrast, a subject-emotional framework is preferred in scenarios with weak negative sentiment and incomplete initial media reporting, or when high media participation coincides with highly sensitive event types. According to these findings, issues of attribution inertia and post-event empathy in government social media responses were identified and the application of public opinion ecosystem governance principles were advocated to enhance dynamic balance, openness, and foresight, thereby optimizing capabilities in public opinion regulation, deep communication, and proactive “preventive care.”

## 1 Introduction

In the contemporary digital landscape, the role of government new media has become increasingly prominent in the public opinion ecosystem. Government agencies have become an important force in guiding public opinion by utilizing new media channels to disseminate information and respond to public opinion incidents (Yuan et al., 2023). However, in the highly transparent network era, the online public opinion ecosystem is fraught with challenges such as information asymmetry, normative deficiencies in public opinion, and a lack of effective guidance (Howard and Parks, 2012). These challenges are exacerbated by the inappropriate response of government new media to public sentiment, which may trigger negative impacts and damage the government’s image and social stability. Therefore, enhancing the capacity of government new media to respond to public sentiment has become a crucial issue in public opinion governance.

Despite the growing importance of government new media, previous studies mainly focus on the specific content of their responses to public sentiment while neglecting the underlying patterns and the broader context of network governance. As representatives of government in the digital realm, government new media combine the communication attributes of new media with the responsibility to maintain government legitimacy (Medaglia and Zheng, 2017). Their response frameworks reflect the patterns of government responses to online public sentiment events. However, these patterns and their integration into the broader field of network governance have not been explored to date. There is lack of a clear stream of literature on the role of these frameworks in regulating public opinion and maintaining government credibility.

In this study, this gap was addressed by examining the response frameworks of government new media from the perspective of public opinion ecology governance. Specifically, fuzzy-set Qualitative Comparative Analysis (fsQCA) was performed on a dataset of 21 public opinion incidents to explore the factors influencing the response frameworks of government new media and to analyze the combination paths of different response frameworks.

By identifying the patterns and mechanisms of public sentiment responses, this study contributes to the theoretical understanding of the interaction between government communication and public opinion in the digital age. Moreover, there are significant practical implications of this study since it offers actionable insights for government agencies to optimize their communication strategies and enhance public trust.

## 2 Literature review and theoretical perspective

### 2.1 The dual role of government social media in public participation

Contemporary research on government social media reveals its evolving dual function as both a crisis communication tool and a participatory governance platform. As demonstrated in the consensus in crisis communication literature demonstrates, government social media facilitates real-time information dissemination during emergencies (Bekkers et al., 2013; Park et al., 2015), while empirical studies on South Korea’s COVID-19 responses further validate its trust-building capacity through transparent crisis updates (Mansoor, 2021; Bae et al., 2023).

On this basis, participatory governance studies have emphasized the transformative potential of social media in enabling bidirectional policy dialogs. A study systematically identified how citizen-government interactions on these platforms enhance policy responsiveness (Lu et al., 2016). This finding has also been corroborated by longitudinal analyses of e-government systems (Chan and Pan, 2008). Notably, Criado et al. (2013) proposed a paradigm shift from information broadcasting to collaborative governance, arguing that platforms such as Twitter can reconfigure traditional power dynamics in policy-making.

However, critical gaps persist between theoretical potential and practical implementation. This discrepancy highlights an under-explored theoretical frontier: how institutional constraints and technological revelations work together to shape the evolution from crisis management to democratic participation. Current research has primarily examined technological capabilities or governance outcomes (Xie, 2020), and the mediating mechanism between platform characteristics and participatory efficacy under-theorized.

This study aims to address these gaps via analyze the following questions:

(1) How do institutional constraints influence the implementation of participatory governance through government social media? and (2) How do the response frameworks of government new media influence the regulation of public opinion and the maintenance of government credibility in the digital age?

### 2.2 Ecological governance of public opinion

An ecological framework was adopted to analyze public opinion governance, a choice grounded in the systematic and dynamic interdependencies revealed by prior literature. It has been reported that the formation and governance of public opinion in the digital era reflect an ecological interplay of technology, power, and information. Wong (2006) uncovered the triadic interaction among social media, public sentiment, and government responses. Casero-Ripollés and Micó-Sanz (2022) established an evolutionary model of digital information ecology through event-driven analysis. These studies transcend linear analytical paradigms, and converge on a tripartite dialectic of “technological empowerment–power restructuring–information evolution,” requiring a framework that systematically deconstructs this complexity.

The dynamics of public opinion and its management were analyzed from the perspective of public opinion ecological governance with the help of an ecological framework guided by ecological theory and the five principles of media ecology (Zuckerman, 2021). Systemic principles emphasizing balance, dynamics, and openness underlie this framework. The media ecology concepts of wholeness, interaction, balance, circulation and resourcefulness were incorporated to provide a comprehensive analysis of media development in this ecosystem (Kostovska et al., 2020). Wholeness recognizes the interconnectedness of the government, the public, and media platforms as an organic system. Interactivity highlights the cyclical feedback loops between technologies, regulations, and social practices. Equilibrium is maintained in information flows, power relations, and value negotiations. Circulation maps the metabolism of information through public opinion cycle and attention shifts. Resourcefulness views information as a contested resource in the process of production, distribution and regeneration. In addition, it promotes an ecological approach characterized by openness, dynamism and vision, emphasizing the interdependence and dynamic interplay of elements in the opinion ecosystem, as well as the need for inclusive information access and proactive strategies to address future challenges.

### 2.3 Media framework

Goffman’s conceptualization of framing—as mutable cognitive schemata that mediate the perception and interpretation of sociocultural phenomena—provides a foundational lens for analyzing discursive practices. In the field of communication, frame theory is divided into two paradigmatic orientations: the media frame, which examines institutionalized narrative structures, and the audience frame, which questions heterogeneous public reception. Seminal analyses of political communication, particularly Semetko and Valkenburg’s (2000) taxonomy of Western media frames, delineate five prototypical frameworks: responsibility attribution, conflict amplification, human interest personalization, economic consequentialism, and moral valuation.

Previous studies on government responses to public opinion predominantly include micro-level case studies on social media usage, neglecting broader analyses of governmental social media responses to significant public events and the overarching frameworks governing such responses. Government social media plays the role of government spokesperson in the digital realm, retaining inherent new media characteristics while assuming governmental responsibilities. Therefore, this study examined the characteristics of government social media in the context of government functions and new media attributes. Existing government response and new media frameworks were synthesized via analyzing government responses to selected high-profile public opinion events. Context-Responsibility and Subject-Emotional were selected as the analysis focus.

Situational framework provides a fine-grained, event-specific process analysis, while the responsibility framework assigns responsibility to individuals, organizations or governments. The Context-Responsibility framework integrates these two perspectives.

Thematic framework provides a macro-level contextualization and offers broader historical and social insights beyond the specific event. Affective framework can be used to analyze emotional interactions with online users and reflects the empathic dimension of the response. The subject-emotional framework combines these two approaches (Table 1).

**Table 1:** Government public opinion response discourse framework classification paraphrase table.

Framework category	Description
Situational framework	Detailed, chronological account of an event, focusing on the process and sequence of actions
Responsibility framework	Ascribes accountability for events to specific actors (individuals, organizations, or governments)
Context-responsibility framework	Integrates situational detail with broader responsibility assessments, linking micro-level actions to macro-level consequences
Thematic framework	Provides a macro-level contextualization of events, incorporating historical, social, and environmental factors beyond the immediate event itself
Affective framework	Analyzes emotional responses and empathetic engagement, especially in online communication. Especially in online communication
Subject-emotional framework	Examines the intersection of subject matter and emotional responses, pairing broad societal context with emotional responses

## 3 Research context

This study examined the role of government social media in public opinion governance within the Chinese context. This study was performed from December 7, 2018, to March 21, 2023, coinciding with significant policy changes and advancements in digital governance. Cases were selected in accordance with the principles of maximum similarity and difference to ensure a diverse and representative sample. The cases were obtained from the “Hot Events” section of the People’s Daily online public opinion channel, which is a platform that aggregates and showcases public opinions and discussions on various social and political issues. This section serves as an important venue for citizens to express their views and for the government to gage public sentiment and reflects a range of public sentiment events, including public security, social conflict events, public management, and enterprise public opinion.

Chinese context presents unique characteristics that influence public opinion governance. The rapid development of digital infrastructure and the widespread adoption of social media platforms have changed the way public sentiment is expressed and managed. Government social media platforms such as Weibo play a dual role in disseminating official information and engaging with public concerns. This dual function creates a complex interplay between government power and public engagement that requires nuanced and adaptive communication strategies.

Although the findings provide valuable insights into the governance of public opinion in China, they are subject to specific boundary conditions. China’s unique socio-political environment, cultural norms and regulatory framework may limit the direct applicability of these findings in other contexts. For instance, the centralized nature of the Chinese government and the emphasis on social stability shape the priorities and approaches in public opinion governance. Additionally, the regulatory environment, including strict oversight of online content and the promotion of positive social values, can influence the types of public sentiments that are prioritized and how they are addressed. These unique conditions may lead to different outcomes in other research settings where such regulatory and cultural contexts do not exist. Nevertheless, the challenges and strategies identified provide broader insights for understanding the role of government social media in global opinion governance.

## 4 Research design

### 4.1 Method

Qualitative Comparative Analysis (QCA), a method developed by Charles C. Ragin in the 1980s, was employed to analyze causal relationships using small sample data. Unlike traditional case studies, QCA combines qualitative and quantitative approaches to explore complex causal configurations and focuses on conjunctural causation rather than simple correlations. Fuzzy-set QCA (fsQCA) was specifically utilized here due to its applicability to analyzing the complex, multi-causal nature of government social media responses to high-profile public opinion events. This approach allows factors to be represented as fuzzy sets, enabling logical operations to capture interactions and influences that cannot be captured by linear quantitative methods.

The methodological implementation follows four sequential steps. First, variable selection was guided by theoretical relevance to public opinion governance frameworks and China’s institutional context. Variables were operationalized using quantifiable indicators to ensure transparency and analytical tractability while capturing critical dimensions of online discourse dynamics. Second, data processing involved a two-stage calibration protocol. Categorical variables were transformed into fuzzy sets, and continuous variables were anchored to the dataset mean with membership thresholds defined by standard deviations. This approach striked a balance between granularity and objectivity, avoiding oversimplification and minimizing subjective bias. Third, a one-conditional necessity analysis was performed to identify potential necessary conditions for the results. Finally, truth table construction systematically mapped all possible combinations of conditions to outcomes.

### 4.2 Research framework

According to the 54th Statistical Report from the China interNet Network Information Center (September 2024), more than 160,000 government Weibo accounts have been verified, and greatly exceed the number of government WeChat accounts. Given the extensive influence of Weibo and their established role as a major source of online public opinion, characterized by decentralization, polycentric dissemination, and real-time interaction, this study comprehensively analyzed the factors that influence the social media response framework in the context of public affairs, using government Weibo as a representative case.

From public opinion ecology, public opinion was divided into five parts: subject, object, essence, carrier, and attractor (Wang et al., 2023). Public opinion goes from generation to fading through the combined force of the five elements: subject driving force, object impact force, essence infecting force, carrier display force and attractor pulling force (Chen and Ge, 2014) (Table 2).

**Table 2:** Public opinion five forces concept table.

Concept	Conceptual interpretation
Subject driving force	This power stems from opinion leaders and influencers, who dominate the discourse through agenda-setting and are amplified by secondary dissemination by ordinary Internet users. Its intensity depends on the quality and volume of information disseminated by these leaders
Object impact force	Measuring multi-dimensional involvement across six domains (politics, economy, military, society, education, culture), this force shows stronger impacts for cross-domain events compared to single-domain occurrences
Essence infecting force	Derived from opinion convergence through multi-granularity sentiment analysis, this force facilitates the transition from disorder to order, aiding event resolution
Carrier display force	Representing the capacity for information dissemination through diverse media platforms, this force enhances display effectiveness through multi-channel propagation
Attractor pulling force	Reflecting the inherent capacity of event to attract social attention, this force is intensified by temporal relevance and multi-event associations (single, serial, clustered, or hybrid events), highlighting its ability to draw public focus

The ecological model of Weibo public opinion was deconstructed into three dimensions: objective existence, relational association, and the nature of motion, which corresponded to the super-network structure, multi-dimensional atlas, and communication mechanism, respective. The super-network structure encompassed the social sub-network, content sub-network, and emotional sub-network, while the multi-dimensional atlas included the subject atlas, theme atlas, topic atlas, and emotional atlas (Wang et al., 2021).

**Figure 1:**
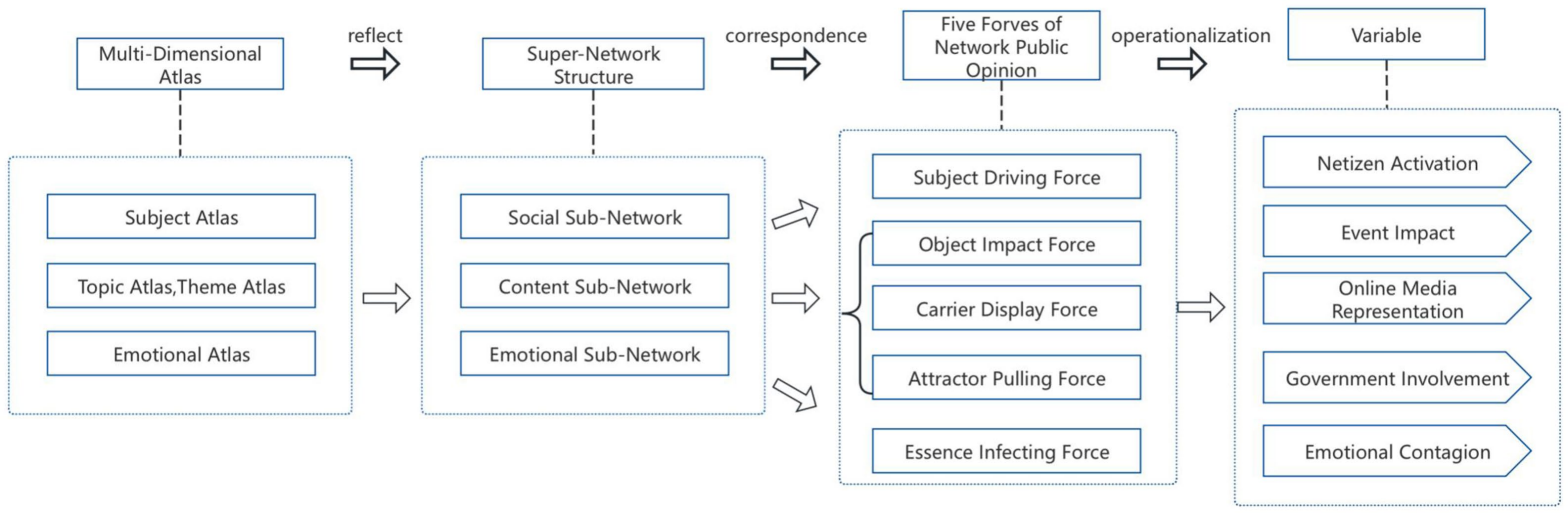
Research framework of the influencing factors of government social media response framework from the perspective of public opinion ecological governance.

Subject atlas reflects the social sub-network corresponding to the subject driving force, and its operationalization is manifested as netizen activation. The topic atlas and theme atlas reflect the content sub-network corresponding to object influence, carrier representation, and attractor traction, which are operationalized as event impact, online media representation, and government involvement, respectively. The emotional atlas reflects the emotion sub-network corresponding to essential infection, which is operationalized as emotional contagion (Figure 1).

The framework first deconstructs multi-agent interactions across the public opinion cycle, synthesizing leadership influence, cross-domain event diffusion, affective self-organization, media effectiveness, and attention dynamics. Second, it structurally unifies ecological patterns via multidimensional mapping and supernetwork analysis, bridging spatial–temporal evolution. Third, it generates operational utility by transforming ecological abstractions into diagnostic intervention tools through quantifiable metrics for multi-stakeholder governance coordination and adaptive resource allocation. Through synergistic mechanism analysis, structural integration, and implementable translation, the framework provides a holistic solution to contemporary e-government challenges in opinion management.

### 4.3 Case selection

Here, Qualitative Comparative Analysis (QCA), guided by the principles of maximum similarity and maximum difference, was adopted to select cases for meaningful comparison. The sample was drawn from the “Hot Events” section of the People’s Daily online public opinion channel. Cases were selected during December 7, 2018 and March 21, 2023. The first date marks the issuance of the “Opinions on Promoting the Healthy and Orderly Development of New Government Affairs Media” by the “General Office of the State Council of the People’s Republic of China.” This policy dramatically changed the way official online public communications are conducted and strengthened the accountability of government entities for online public opinion events. The second date signifies the “Sharing, Creating, Common Benefit” summit, a collaborative effort involving Weibo and key stakeholders. This event highlighted the growing sophistication and influence of government Weibo and demonstrated a significant shift in government engagement on social media.

Initial case was selected considering public opinion intensity and prominent online search rankings. Cases without apparent government Weibo response were excluded. The final selection prioritized diversity, including various event types categorized by People’s Daily Online. Selected events had key characteristics: non-political nature, unexpected occurrence, rapid dissemination, negative impact, and potential replicability (Table 3).

**Table 3:** Hot public opinion event cases.

Order number	Case	Time	Event type
1	The disappearance of a middle school student named Hu Xinyu has attracted attention	2022.11.19–2023.2.3	Public security
2	Hebei Tangshan vicious wounding case	2022.6.10–8.30	Public security
3	The mathematics textbook of human education edition cited controversy	2022.5.26–8.23	Enterprise public opinion
4	“Sky-high” funeral fees lead hot discussion	2022.4.10–4.12	Social contradictions
5	Fengxian birth eight children woman incident	2022.1.28–2.25	Public management
6	Many places across the country “pull power cuts” attracted attention	2021.10.6–10.8	Public management
7	The policy of “double reduction” in education has aroused heated social discussion	2021.6.24–8.19	Public management
8	Dark events in tumor treatment	2021.4.19–5.7	Public security
9	The “Bride price loan” has caused social controversy	2021.3.16–4.7	Social contradictions
10	A female user jumped out of the car	2021.2.22–3.3	Enterprise public opinion
11	Dunhuang ten thousand mu of shelterbelt was destroyed incident	2021.1.20–1.29	Public security
12	The rise of community group-buying business attracts attention	2020.2.25–2021.1.3	Public administration
13	Primary school students have won cancer awards	2020.7.5–7.17	Social contradictions
14	The release of the “Street Vendor Economy” has triggered a hot discussion	2020.5.14–6.8	Public management
15	Feng Chao express cabinet charges lead to hot discussion	2020.4.29–5.19	Enterprise public opinion
16	The first case of face recognition has attracted public attention	2019.11.2–2020.11.30	Public security
17	Xiangxi female teacher Li Tiantian issued to attract attention	2019.10.17–10.25	Social contradictions
18	Shanghai subway is the first “salty pig sexual harassment” into punishment	2019.8.11–10.15	Public security
19	The “college entrance examination immigration” incident in Shenzhen has attracted close attention	2019.4.30–5.20	Social contradictions
20	Zhai Tianlins fake paper has sparked heated discussion	2019.2.8–2.19	Public character
21	Wenzhou underage “female virtue class” caused a hot discussion	2018.12.8–12.12	Public administration

### 4.4 Variable setting and data processing

#### 4.4.1 Variable setting

During the variable selection, a multifaceted approach integrating QCA’s research framework, theoretical perspectives, and literature review was employed. To keep the analysis manageable with a moderate sample size, the number of conditions was limited to seven. Combining the Five Forces Model of public opinion and the panoramic model of Weibo mentioned above, the following conditional variables were identified: netizen attention, online media participation, event type, level of government intervention, and proportion of negative sentiment.

Netizen attention was measured by using Weibo hot search rankings from Qingbo that a leading Chinese new media big data platform. The influence of key opinion leaders was assessed by adding the number of followers of the top three most influential users on the Weibo “Hot” channel for each event.

Online media participation was quantified using Baidu Index information scores, reflecting online news coverage and attention.

Information completeness in initial reporting was categorized into four levels (open and complete, largely complete, incomplete, and absent) based on Qingbo data and existing literature.

Event type was classified (public management, social contradictions, public security, and enterprise public opinion) using a sensitivity-based weighting scheme.

Government intervention level was categorized according to the standard administrative hierarchy (central, provincial, municipal, county/township).

Finally, negative sentiment was measured using the proportion of negative user comments derived from Qingbo’s sentiment analysis tool.

Government social media response frame focuses on details and specific event processes, has distinct rule content in selected cases, and is set as a context-responsibility framework. In addition, the government social media response frame has empathic content emphasizes macro-descriptions, and is set as a subject-emotional framework (Table 4).

**Table 4:** Description of the assignment of each variable.

Variable type	Variable	Secondary indicator variable and coding	Variable value assignment and description
Condition variable	Netizen attention	Attention degree of Netizen (AN)	Weibo hot search on the list, mean anchor
		Opinion Leader communication (OL)	The sum of the number of the fans and the average anchor point
Condition variable	Online media participation	The degree of participation of online media (MP)	Baidu index information index daily average, mean anchor point
		Information completeness in initial reporting (IC)	The complete value of public information is 1, the basic complete value of disclosed information is 0.67, the completeness of disclosed information is 0.33, and basically no public information is 0
Condition variable	Event type	Event type sensitivity (ET)	According to the weight of sensitive information, the values of public management type, social contradiction type, public security type and enterprise public opinion are 1, 0.67, 0.33, and 0, respectively
Condition variable	Government intervention	Government intervention level (GL)	The central assignment was 1, the provincial level 0.67, the municipal level 0.33 and the county and township level 0
Condition variable	Negative sentiment	Proportion of negative information (NI)	Negative information ratio, the mean anchor point
Outcome variable	Response discourse framework	Context-responsibility framework (DF1)	Government affairs new media response Context-Responsibility framework assigned value is 1, no assigned value is 0
		Subject-emotional framework (DF2)	Government affairs new media response to the emergence of the theme of the emotional framework is assigned a value of 1, does not appear to be assigned a value of 0

#### 4.4.2 Data calibration

The calibration of fuzzy sets involved assigning partial membership scores between “0” and “1” to assess the degree of membership of a condition between “complete non-membership” and “complete membership.” In this work, the “Four-Value Fuzzy Set Calibration Method” and the “Mean Anchor Method” were used for condition variables. “Four-Value Fuzzy Set Calibration Method” involved a quartile equal interval calibration based on the degree of membership of cases in the variable, with the values [1-0.67-0.33-0] assigned accordingly. Scores of “1” and “0”indicate complete membership, and complete non-membership, respectively. For the “Mean Anchor Method,” the mean value was used as an anchor point to divide the variable into four parts, with values assigned of [1-0.67-0.33-0]. The outcome variable was assigned by a binary method.

#### 4.4.3 Necessity analysis of single condition variable

In Qualitative Comparative Analysis (QCA), the necessity analysis of single condition was determined by the consistency indicator, which measures the explanatory power of a single condition variable on the outcome variable. When the consistency indicator is greater than 0.8, the single condition is considered sufficient for the outcome. If the consistency indicator exceeds 0.9, the single condition is deemed a necessary condition for the outcome. The results of the necessity analysis of the single condition variable were obtained by performing calculations using the fuzzy set qualitative comparative analysis (fsQCA) software.

As shown in Table 5, no single condition variable can serve as a necessary condition when using the context-responsibility framework as the outcome variable. Given that the NIMBY outcome is characterized by “multiple complex concurrent causalities,” further analysis of combinations of condition variables was conducted to gain novel insights.

**Table 5:** Single condition necessity analysis of context-responsibility framework (DF1) and ~ context-responsibility framework (~DF1).

Analysis of necessary conditions				
Conditions tested:				
Outcome variable: DF1			Outcome variable: ~DF1	
	Consistency	Coverage	Consistency	Coverage
AN	0.479167	0.603358	0.420000	0.396642
~AN	0.520833	0.544900	0.580000	0.455100
OL	0.485000	0.668965	0.320000	0.331034
~OL	0.515000	0.502439	0.680000	0.497561
MP	0.353333	0.543590	0.395556	0.456410
~MP	0.646667	0.587879	0.604444	0.412121
IC	0.498333	0.642320	0.370000	0.357680
~IC	0.501667	0.514970	0.630000	0.485030
ET	0.555833	0.513077	0.703333	0.486923
~ET	0.444167	0.666250	0.296667	0.333750
GL	0.499167	0.486596	0.702222	0.513404
~GL	0.500833	0.691600	0.297778	0.308400
NI	0.661667	0.786139	0.240000	0.213861
~NI	0.338333	0.372477	0.760000	0.627523

As shown in Table 6, no single condition variable can be considered necessary when the subject-emotional framework serves as the outcome variable. Considering that the NIMBY results are “multiple complex concurrent causal relationships,” the combination of condition variables must be further analyzed to obtain more comprehensive information.

**Table 6:** Single condition necessity analysis of subject-emotional framework (DF2) and ~ subject-emotional framework (~DF2).

Analysis of necessary conditions				
Conditions tested:				
Outcome variable: DF2			Outcome variable: ~DF2	
	Consistency	Coverage	Consistency	Coverage
AN	0.403333	0.634837	0.580000	0.365163
~AN	0.596667	0.780296	0.420000	0.219704
OL	0.340667	0.587356	0.598333	0.412644
~OL	0.659333	0.804065	0.401667	0.195935
MP	0.354667	0.682051	0.413333	0.317949
~MP	0.645333	0.733333	0.586667	0.266667
IC	0.376667	0.606874	0.610000	0.393126
~IC	0.623333	0.799829	0.390000	0.200171
ET	0.666667	0.769231	0.500000	0.230769
~ET	0.333333	0.625000	0.500000	0.375000
GL	0.621333	0.757108	0.498333	0.242892
~GL	0.378667	0.653625	0.501667	0.346375
NI	0.415333	0.616832	0.645000	0.383168
~NI	0.584667	0.804587	0.355000	0.195413

## 5 Results

According to the requirements of the QCA methodology, as a small sample study, the minimum case frequency was set to 1 and the consistency threshold was set to 0.8 when constructing the truth table to ensure the explanatory strength of the configurations. In configuration path analysis, the solution consistency is higher than or equal to 0.8 to prove that the solution is valid. After calculating the configuration paths using the fsQCA software, the intermediate solution was reported as the optimal solution to provide data to support the conclusions.

### 5.1 Results of the context-responsibility framework

#### 5.1.1 Construction of the truth table

The true value table of the context-responsibility framework (DF1) shows that all 21 cases can fall on the 14 combined variables, confirming the credibility of the condition combination (Table 7).

**Table 7:** The truth table of the context–responsibility framework (DF1).

Attention degree of netizen	Opinion leader communication	The degree of participation of online media	Information completeness in initial reporting	Event-type sensitivity	Government intervention level	Proportion of negative information	The number of cases	Context-responsibility framework
1	1	1	0	0	0	1	1	1
1	1	0	0	1	0	1	1	1
1	1	0	1	1	0	1	1	1
0	1	1	1	0	1	1	1	0
0	1	0	1	1	0	1	1	0
1	0	0	1	1	0	0	1	0
0	0	0	0	1	0	0	1	0
0	0	1	0	1	0	1	1	0
0	0	0	0	0	1	1	1	0
0	1	0	0	1	1	0	1	0
1	0	1	0	1	0	0	1	0
1	0	0	0	1	1	0	1	0
0	1	1	0	1	0	0	1	0
1	0	1	0	1	1	0	1	0

#### 5.1.2 Configuration analysis

The consistency of the intermediate solution of the context-responsibility framework is greater than 0.8, indicating that this solution is valid, and three configuration paths are derived (Table 8):

**Table 8:** Configuration analysis and configuration combination of context-responsibility framework.

Variable	Path 1	Path 2	Path 3
AN	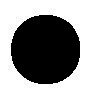	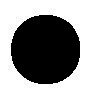	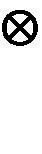
OL	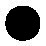	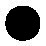	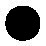
MP	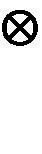	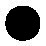	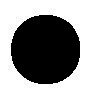
IC		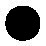	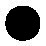
ET	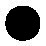	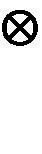	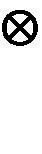
GL	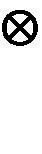	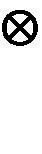	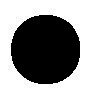
NI	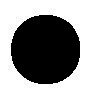	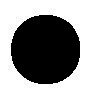	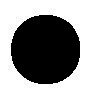
Raw coverage	0.160833	0.1225	0.114167
Unique coverage	0.0766667	0.0333333	0.0591667
Consistency	0.861607	0.967105	0.805882
Solution coverage	0.2625		
Solution consistency	0.842246		

Path 1: Netizen Attention*Opinion Leader Influence* ~ Online Media Participation*Negative Information*Event Type* ~ Government Intervention, with Netizen Attention and Negative Information as the core conditions.

Path 1 indicates that with a high netizen attention, opinion leader influence, negative information, and sensitive event types exist, the higher the netizen attention and the larger the proportion of negative information, the more likely it is that the government’s social media response framework will become a context-responsibility framework.

Path 2: Netizen Attention*Opinion Leader Influence*Online Media Participation*Negative Information* ~ Initial Reporting Information Completeness* ~ Event Type* ~ Government Intervention, with Netizen Attention and Negative Information as the core conditions.

Path 2 implies that when there is high netizen attention, influence of opinion leaders, online media participation, and negative information, the higher the netizen attention and the greater the proportion of negative information, and the more likely the response framework of government social media is to be a context-responsibility framework.

Path 3: ~Netizen Attention*Opinion Leader Influence*Online Media Participation*Negative Information*Initial Reporting Information Completeness* ~ Event Type*Government Intervention, with Online Media Participation, Negative Information, and Government Intervention as the core conditions.

Path 3 suggests under the influence of opinion leaders, online media participation, negative information, complete initial reporting information, and government intervention, the higher the online media participation, the greater the proportion of negative information, and the higher the level of government intervention, and the more likely the response framework of government social media is to be a context-responsibility framework.

### 5.2 Results of the subject-emotional framework

#### 5.2.1 Construction of the truth table

In accordance with the true value table of the subject-emotional framework (DF2), all 21 cases can fall on the 14 combined variables, confirming the credibility of the condition combination.

#### 5.2.2 Configuration analysis

The consistency of the intermediate solution of the subject-emotional framework higher than 0.8, indicating that this solution is valid, and six configuration paths are derived (Table 9):

**Table 9:** The truth table of the subject-emotional framework (DF2).

Attention degree of netizen	Opinion leader communication	The degree of participation of online media	Information completeness in initial reporting	Event-type sensitivity	Government intervention level	Proportion of negative information	The number of cases	Subject-emotional framework
0	0	0	0	1	0	0	1	1
0	0	1	0	1	0	1	1	1
0	1	1	0	1	0	0	1	1
1	0	0	0	1	1	0	1	1
0	1	0	0	1	1	0	1	1
1	0	1	0	1	1	0	1	1
1	0	1	0	1	0	0	1	1
0	0	0	0	0	1	1	1	0
1	1	0	0	1	0	1	1	0
0	1	1	1	0	1	1	1	0
1	0	0	1	1	0	0	1	0
0	1	0	1	1	0	1	1	0
1	1	0	1	1	0	1	1	0
1	1	1	0	0	0	1	1	0

Path 1: Netizen Attention * ~ Opinion Leader Influence * Media Participation * ~ Initial Reporting Information * Event Type * ~ Negative Information. Media participation, ~initial reporting information, event type, and ~negative information are the core conditions.

Path 2: Netizen Attention * ~ Opinion Leader Influence * ~ Initial Reporting Information * Event Type * Government Intervention * ~ Negative Information. ~Initial reporting information and ~negative information are the core conditions.

Path 3: ~ Netizen Attention * ~ Opinion Leader Influence * ~ Media Participation * ~ Initial Reporting Information * Event Type * ~ Government Intervention * ~ Negative Information. ~ Initial reporting information and ~negative information are the core conditions.

Path 4: ~ Netizen Attention * Opinion Leader Influence * Media Participation * ~ Initial Reporting Information * Event Type * ~ Government Intervention * ~ Negative Information. Media participation, ~initial reporting information, event type, and ~negative information are the core conditions.

Path 5: ~ Netizen Attention * ~ Opinion Leader Influence * Media Participation * ~ Initial Reporting Information * Event Type * ~ Government Intervention * Negative Information. Media participation and event type are the core conditions.

Path 6: ~ Netizen Attention * Opinion Leader Influence * ~ Media Participation * ~ Initial Reporting Information * Event Type * Government Intervention * ~ Negative Information. ~Initial reporting information and ~negative information are the core conditions.

In accordance with the conventions of fsQCA, only intermediate solutions require detailed explanation. The six paths identified in this study are intermediate solutions. However, given the complexity of path combinations, the parsimonious solutions were compared and found that Paths 3 and 6 both contained missing core conditions and exhibited high overlap, rendering them non-representative of the overall results. Path 4 shared identical core conditions with Paths 1 and 5, and thus was subsumed under these paths. Therefore, Paths 1, 2, and 5 were discussed in detail.

Path 1 indicates that in situations where there is high netizen attention, media participation, and sensitive event types, the higher the media participation, the lower the completeness of initial reporting information, and the lower the proportion of negative information, and the more likely the response framework of government social media is to be a subject-emotional framework.

Path 2 indicates that when there is high netizen attention, sensitive event types, and government intervention, the lower the completeness of initial reporting information and the lower the proportion of negative information, and the more likely the response framework of government social media is to be a subject-emotional framework.

Path 5 indicates that when there is media participation, sensitive event types, and the presence of negative information, the higher the media participation and the more sensitive the event type, and the more likely government social media is to respond using a subject-emotional framework (Table 10).

**Table 10:** Configuration analysis and configuration combination of subject-emotional framework.

Variable	Path 1	Path 2	Path 3	Path 4	Path 5	Path 6
AN	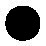	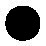	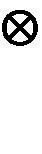	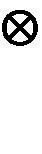	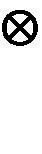	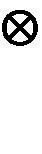
OL	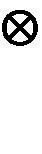	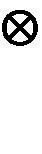	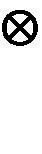	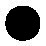	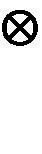	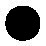
MP	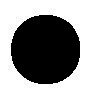		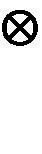	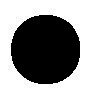	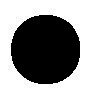	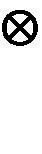
IC	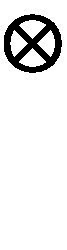	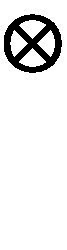	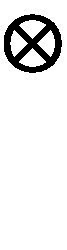	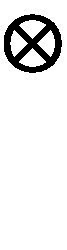	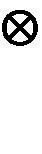	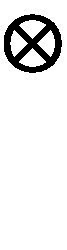
ET	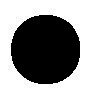	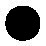	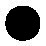	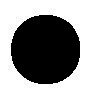	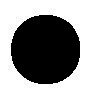	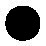
GL		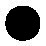	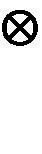	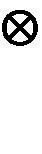	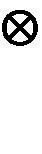	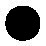
NI	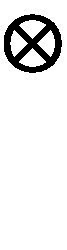	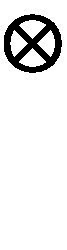	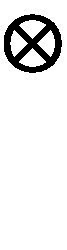	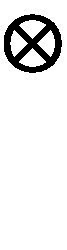	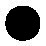	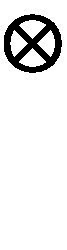
Raw coverage	0.136	0.174	0.148	0.0993333	0.0886667	0.135333
Unique coverage	0.012	0.0366667	0.0413333	0.0133334	0.03	0.016
Consistency	0.910714	0.928826	0.961039	0.93125	0.93662	0.918552
Solution coverage	0.335333					
Solution consistency	0.958095					

### 5.3 Robustness test

In order to avoid the randomness and sensitivity of the results, the robustness test method of fsQCA was used to eliminate some cases for analysis. Specifically, leave-one-out cross-validation was performed by iteratively excluding three boundary cases (Cases #7, #15, #21) and re-running the analysis. This process was repeated for each of the 21 public sentiment events, resulting in 21 subsamples. The core configurational conditions identified in the initial analysis remained consistent across all iterations, with Configuration 1 appearing in 18 out of 21 subsamples (85.7%). The results show that the data analysis conclusions are robust.

## 6 Conclusion

Government social media platforms usually employ a context-responsibility framework in scenarios characterized by high public attention, strong negative sentiment, or significant involvement from media and government entities. In contrast, a subject-emotional framework is favored when negative sentiment is minimal, initial media reports are incomplete, or media attention is heightened for sensitive events. These findings emphasize predictable patterns of government social media responses to online public opinion and highlight the challenges in information processing and communication. Two notable limitations were identified: “attribution inertia” and “post-event empathy.”

Attribution inertia refers to the excessive reliance on the context-responsibility framework in complex situations, which may intensify public dissatisfaction. Attribution inertia manifests through bureaucracies’ overreliance on context-responsibility frameworks to manage high-stakes public opinion events. In the case of “Bride Price Loan,” involving a controversial microfinance product, authorities prioritized procedural clarification of bank regulation while avoiding substantive engagement with underlying gender issues, a pattern that reflects an institutional decoupling between administrative protocols and the reconstruction of sociocultural discourse. Similarly, the “Underage Female Virtue Class” case revealed the paradoxical results of hierarchical interventions: despite swift regulatory action against unethical educational practices, strict adherence to the accountability narrative exacerbates public skepticism about systemic governance failures rather than addressing legitimacy deficits. These cases demonstrate how standardized response frameworks become tools for performative risk containment rather than adaptive problem-solving. Further, attribution inertia epitomizes the transformation of bureaucratic risk calculations into networked environments, where standardized frameworks act as rituals for maintaining legitimacy rather than as tools for genuine engagement.

Post-event empathy pertains to delayed emotional responses that lack timeliness and proactive engagement, potentially undermining credibility. The limitations of post-event empathy emerge through temporal dislocations in emotional engagement strategies. The “Street Vendor Economy” policy rollout illustrates critical temporal misalignment—empathic narratives about economic inclusivity emerged 72 h post-announcement, missing the 6-h window when public confusion peaked. Spatial fragmentation compounded these issues in the “Shanghai Metro Sexual Harassment Ruling” case while combining legal rigor with victim solidarity rhetoric on Weibo, official accounts maintained a detached legalistic tone, thereby creating an empathy asymmetry unique to the platform. This disjointed communication reflects an underdeveloped temporal thematic coordination. Furthermore, Post-event empathy deficiencies expose the temporal rigidity of state affective labor, in contrast to the real-time fluidity of the digital emotional landscape.

This study extends prior research by detailing the media frameworks used by government social media in response to public opinion events. Our findings identify specific response mechanisms, i.e., attribution inertia and post-event empathy. These insights refine existing research on the disconnect between public demands and government discourse in China and provide a more nuanced understanding of how government social media manages public opinion events.

Although this study was carried out situated within the Chinese context, the identified challenges are relevant to other countries using government social media platforms. Attribution inertia and post-event empathy illustrate the pitfalls of overreliance on standardized frameworks, suggesting that governments should develop more adaptive and responsive communication approaches. The need for adaptive and timely communication strategies is universal, and our findings provide valuable insights for improving public engagement through social media globally.

## 7 Suggestion

In order to enhance the regulatory and moderating function of government social media in shaping public opinion, it is necessary to maintain a dynamic balance in the platforms, and between the platforms and their external environments. This dual circulation model is crucial for the ongoing support and development of government social media. In order to accomplish this goal, an integrated framework will be implemented using a holistic strategy across multiple dimensions.

First, government social media platforms must enhance their responsiveness and efficacy in addressing public opinion by serving as a barometer for new media sentiment and curbing the spread of negative discourse (Li et al., 2021). Therefore, AI-driven dynamic response systems that integrate real-time sentiment monitoring with adaptive frameworks should be deployed (Al-Mushayt, 2019). Additionally, media activities should be guided and supervised to ensure objective and impartial reporting. Response strategies should be developed to address underlying causes and provide solutions, rather than relying on formulaic methods.

Secondly, transparency plays an essential role in the effective governance of the public opinion ecosystem (Lorenz-Spreen et al., 2020). For government-operated social media platforms, multifaceted approach must be adopted to prioritize stakeholder engagement and data accessibility. Specifically, platforms must actively engage with diverse stakeholders through social, content, and emotional networks, prioritizing deep communication that establishes emotional connections beyond mere information dissemination (Leppert et al., 2022). This can be achieved by employing networked communication strategies and utilizing the social vernacular of new media. On the other hand, open-access query systems should be developed to retrieve historical case-handling records and decision-making criteria through keyword searches for citizens (Bertot et al., 2012). For example, detailed records of resolved cases since 2021 can be obtained by searching for “Bride Loan.” This combination of deep communication and transparent data presentation will highly enhance public trust and effective governance.

Finally, the management of government social media necessitates a substantial degree of foresight to effectively comprehend and navigate online public opinion. A strategic shift from reactive to proactive engagement is imperative and focuses on addressing issues at their origin (Liu and Zhang, 2018). By identifying accountable entities and understanding the fundamental patterns in the evolution of online public sentiment, government social media platforms can implement “preventive measures” to preclude the escalation of public sentiment. This proactive “preventive measures” enhances their ability to manage and mitigate potential issues before they manifest.

In conclusion, it is crucial to maintain a dynamic equilibrium, reinforce transparency, and advance strategic foresight to augment the efficacy of government social media in managing public opinion. These strategies will enable government social media to adeptly navigate the intricate and continually evolving landscape of online public opinion.

## 8 Limitation

In this work, a small sample was used. Consequently, the model may not fully encompass all factors influencing the public opinion response of government social media frameworks. Future research should consider expanding the number of cases and variables for a more comprehensive analysis.

## Data Availability

The original contributions presented in the study are included in the article/supplementary material, further inquiries can be directed to the corresponding author.
